# From comfortable to conflicted: a three-year longitudinal symptom evolution of problematic Internet use among junior high school students

**DOI:** 10.3389/fpsyt.2025.1635911

**Published:** 2025-09-05

**Authors:** Yanjun Kang, Xun Feng, Tong Zhang, Kexin Yin, Peng Wang

**Affiliations:** ^1^ School of Psychology, Shandong Normal University, Jinan, China; ^2^ School of Education, Wenzhou University, Wenzhou, China; ^3^ Key Research Center of Philosophy and Social Sciences of Zhejiang Province (Institute of Medical Humanities, Wenzhou Medical University), Wenzhou, China

**Keywords:** problematic Internet use, network analysis, longitudinal study, network comparison, junior high school students

## Abstract

**Background:**

Problematic Internet use (PIU) is frequently regarded as a potential mental health concern and poses significant risks to the well-being of adolescents. Previous studies have predominantly focused on the influencing factors and harms of PIU, with less attention directed towards symptom manifestations and age differences, especially among junior high school students entering adolescence.

**Methods:**

This study used network analysis to explore the core symptoms and symptom relationships of PIU among junior high school students over three years. This study recruited 302 sixth-graders (T1: M=11.6, SD=0.65) who were assessed using the Problematic Internet Use Scale four times, resulting in four networks.

**Results:**

The network analysis showed that at T1, ‘hard to control’ and ‘being conflicted’ had the highest correlation. At T2-T4,’being comfortable’ and ‘Feel bad mood’ exhibited the highest correlation. At T1 and T2, ‘being comfortable’ was the core PIU symptom, reflecting students’ psychological dependence on the Internet in early middle school. However, ‘being conflicted’ becomes the most critical symptom in later middle school as they age, revealing the increasing contradiction between cognition and behaviour. In general, “being comfortable”, “feel bad mood”, and “being conflicted”, which constitute the key nodes in the PIU relationship network, generally maintained high-intensity centrality across the four measurements. Network comparison tests indicated similar network structures and connection strengths across the four measurements, with no significant gender differences.

**Conclusions:**

The current study indicated that in the early stage of junior high school, implementing precise interventions for the symptom of “being comfortable” may help prevent the further deepening of problematic Internet use, while in the later stage of junior high school, close attention should be paid to the problem of “being conflicted” among adolescents. In addition, interventions for problematic Internet use issues do not need to take gender into account. This study provides evidence for understanding the dynamic changes in PIU among junior high school students and serve as a reference for formulating more appropriate and effective prevention and intervention measures.

## 1 Introduction

According to the International Telecommunication Union (ITU) report “Facts and Figures 2024,” the global number of Internet users is expected to reach 5.5 billion by the end of 2024. The internet has become an indispensable part of modern society, and adolescents may be particularly susceptible to the allure of various online activities. (1). In China, according to the ‘53rd China Internet Development Status Report’ released by the China Internet Network Information Centre (2), the proportion of adolescent Internet users nationwide had reached 18.5% as of December 2023. The breadth and depth of Internet usage among minors have significantly increased. Although the Internet can bring convenience to adolescents’ learning and daily lives, excessive use can lead to various adverse effects (3), such as problematic Internet use (PIU) (4, 5). However, there is still no consensus on the terms or definitions of PIU so far. Many studies also describe PIU as Internet addiction, Internet dependence, compulsive Internet use, problematic Internet use, etc (6–8). Generally speaking, these terms are regarded as synonyms (9). As PIU has not yet been classified as a mental disorder by the World Health Organization (WHO), the Diagnostic and Statistical Manual of Mental Disorders (DSM-5), or the International Classification of Diseases (ICD-11), many scholars consider the use of the term “addiction” to be too severe. Therefore, many scholars prefer to use the less controversial term “problematic Internet use”. According to Davis’ (4) cognitive behavioral model, he classified PIU into specific problematic Internet usage and broad problematic Internet usage. The former refers to an individual’s use of specific online Internet activities (for example: Specific subtypes of online game addiction, social media addiction, online gambling, etc. (10). In this study, we define PIU as an maladaptive Internet usage pattern resulting from excessive use of the Internet, smartphones or specific social media.

Adolescence is a critical period for individual physical and psychological development. However, adolescents are in a special period of physical and mental development, in which they show strong curiosity about emerging technologies and weak self-control and discrimination skills (11). Furthermore, academic pressure during this stage often leads adolescents to use smartphones excessively as a means of relaxation, contributing to PIU (41). Of particular concern are junior high school students entering puberty, which is characterised by fragile psychological defences, heightened curiosity, and a strong desire to explore novel experiences. As they face academic pressure and diverse interpersonal challenges, junior high school students are more susceptible to Internet addiction than other age groups and may find it challenging to disengage from the Internet (13). PIU may negatively affect adolescents’ academic performance (14), physical and mental health (15), and interpersonal relationships (16). Furthermore, it can result in maladaptive cognition, non-content-specific behaviors (4), as well as risky (17) and even criminal actions (18). Given the significant adverse effects of PIU on adolescents and their heightened vulnerability (19, 20), Given the high prevalence and significant harm associated with adolescent Problematic Internet Use (PIU), there is an urgent need to expand and deepen the understanding of PIU in adolescents. This will provide new perspectives and approaches for the assessment and early warning of adolescent PIU. Therefore, exploring how PIU symptoms manifest in adolescents is crucial.

Recently, the rapid rise in network analysis methods has provided new perspectives for studying human psychological phenomena and mental disorders (21, 22). Network analysis is an emerging approach for identifying the symptoms of Problematic Internet Use (PIU) and understanding the relationships between them. Symptoms are nodes, and the interactions between symptoms are edges, with the strength of edges represented by the thickness of the connections in a network graph. Network analysis visualises the relationships between symptoms and identifies core symptoms that exhibit high centrality and strong correlations with other symptoms in the network (23, 24). Therefore, identifying core symptoms can help determine the critical mechanisms underlying disease onset and maintenance. Interventions targeting core symptoms are more effective than those targeting peripheral symptoms, potentially preventing symptom exacerbation (25).

Network analysis was initially primarily used to examine psychiatric symptoms. For instance, several studies have utilized network analysis to investigate psychiatric symptoms such as depression (26, 27), autism spectrum disorders (28), and mental illnesses in general (29). As research advances, network models are no longer limited to the analysis of mental disorders. An increasing number of researchers are applying network analysis to issues related to Internet use. For example, Sánchez-Fernández et al. (30) used network analysis to explore the interactions among problematic online behaviour, psychological distress, and emotional health, finding that depression, anxiety and stress were central nodes in the problematic online behaviour–psychological distress–emotional role restriction network. Zhou et al. (31) investigated longitudinal network relationships between symptoms of Internet gaming problems and internalising and externalising issues in early Chinese adolescents, revealing gender-specific pathways influencing depression, irritability, and issues related to Internet gaming through a cross-lagged panel network model.

Adolescents undergo rapid physical and psychological changes during puberty, making it crucial to identify and assess the dynamic variability of PIU in this population (32). Research has found that the prevalence of PIU may vary with age, with early adolescence being a critical period for the onset of issues related to Internet use (33). The prevalence of PIU has increased rapidly, with the highest rates in late adolescence (34). Therefore, investigating specific symptoms during each stage of adolescence is crucial for preventing and intervening in PIU. However, few studies have used network analysis to examine age-related differences in PIU among adolescent. For example, Hirota et al. (35) compared the PIU networks of adolescents with ASD to those of adolescents with typical neurological development, finding that ‘failure to reduce Internet use’ and ‘anticipation of future online activities’ were core symptom expressions in the general adolescent population. Cai et al. (36) conducted a network analysis on central symptoms of Internet addiction and depression among Macanese adolescents, identifying ‘focused on the Internet’, ‘neglecting chores to spend more time online’, ‘guilt’, and ‘requesting extended online time’ as the most central symptoms in the network of Internet addiction. Liu et al. (37) explored the core symptoms and symptom relationships of PIU across different periods of adolescence and found that increased satisfaction time, inability to stop, and feelings of depression were the core symptoms in early, middle, and late adolescence, respectively. However, these studies relied primarily on cross-sectional data. Although Liu et al. (37) conducted a study on PIU in adolescents at different stages of puberty, the comparison was made across three independent samples. The network structure at different developmental stages may not fully reflect the changes in the PIU symptom network of the same individual over time. Therefore, longitudinal data are essential to investigate the evolution of PIU symptom networks across developmental stages. Junior high school students are in the early stages of adolescence and face multiple challenges related to academic, social, and physical development (38). Research focusing on this critical developmental stage could reveal the characteristics and patterns of PIU in this age group. However, no studies to date have conducted a longitudinal network analysis of PIU among adolescents in middle school.

Does PIU exhibit gender differences among adolescent? Recent studies have reported gender differences in Internet-related behaviors (39), such as males being more inclined to gaming, while females tend to engage in gender comparison through the Internet (1). Moreover, male and female Internet addiction behaviors also differ (40). For instance, Wang (12) studied differences in symptom networks of Internet gaming addiction, anxiety, and depression among college students and found the core symptom of Internet gaming addiction was ‘salience’; however, the at-risk group differed by gender, with the core symptom being ‘mood modification’ in men and ‘salience’ in women.This suggests that gender may influence adolescents’ PIU. However, there are also conflicting findings. Some researchers, using latent growth models, have found no significant gender differences in the rate of increase of PIU among adolescents (42). Additionally, Cai et al. (36) and Liu et al. (37), through network analysis, found no significant differences between male and female adolescents in terms of overall Internet usage intensity. These inconsistencies suggest that gender differences may not be reflected in the severity of PIU, but could instead manifest in the structural characteristics of the symptom network. In other words, adolescents of different genders may develop PIU through distinct psychological and behavioral pathways. Therefore, this study posits that there may be significant differences in the structure of problematic Internet use (PIU) symptom networks between males and females, and further systematic research on gender differences in PIU among junior high school students is warranted.

Scholars have pointed out that undirected network analysis, network comparison, and cross-lagged network analysis methods are all applicable to longitudinal data (43). However, they focus on different aspects: the former two emphasize the differences in core symptoms and symptom relationships at different time points, while cross-lagged network models aim to reveal causal relationships of symptoms across time. Current scholars believe that these methods all provide valuable insights (44). Therefore, in line with the objectives of this study, we employ network analysis to explore the evolution of core symptoms of PIU over three years among junior high school students, and use network comparison testing to investigate the differences in network structure and overall connectivity strength across different ages and genders.

## 2 Methods

### 2.1 Participants

The study data were derived from a longitudinal investigation of behavioural issues related to Internet use. The participants were sixth-grade students from Shandong Province, China. Junior high schools in this region follow a three-year program. The study spanned three years and consisted of four assessments aimed at evaluating symptoms of PIU among adolescents, tracking them from sixth to ninth grade. The first assessment was conducted before the sixth-grade midterm examination, followed by subsequent assessments before the midterm examinations in seventh, eighth, and ninth grade, respectively. Initially, teachers were entrusted to distribute a parental informed consent form introducing the study, which parents signed and returned to the teachers. The researchers then collected these forms before conducting the assessments. 302 valid questionnaires were obtained (M=11.6, SD=0.65), comprising 136 boys (45%) and 166 girls (55%). The average participant age at the first assessment was 11.6 years (SD=0.65). Harman’s single factor test was used to test the results, and the results showed that there were 9 factors with characteristic roots greater than 1, and the variation explained by the largest factor was 24.66%, which was less than 40% of the critical standard (45). Therefore, there was no significant common method bias in this study.

### 2.2 Procedure

We employed a group-testing approach organised by class units using questionnaire assessments for data collection. Graduate students studying psychology who had received rigorous professional training as test administrators conducted the assessments. Class teachers were present to assist in maintaining discipline but were not actively involved in the testing process. At the beginning of each assessment, the participants were briefed on the basic requirements of the test. They were informed that they should respond truthfully according to their own situation. Responses were not provided as ‘right’ or ‘wrong’ and were purely for scientific research purposes and unrelated to academic grades. No one except the researchers had access to the participants’ responses.

### 2.3 Research Instrument

PIU was measured using the Problematic Internet Use Scale (PIUS-a) developed by Rial et al. (46). This scale was adapted and developed to assess students’ Internet use-related issues. It comprises 11 items, such as ‘When you are online, time seems to pass quickly’. The PIU-a is scored using a 7-point Likert scale, with 1 indicating ‘strongly disagree’ and 7 indicating ‘strongly agree’. No items are reverse-scored. The total score is obtained by summing the scores for each item. Higher total scores indicate higher levels of PIU. In this study, Cronbach’s α coefficients for the PIU-a were 0.87, 0.87, 0.89, and 0.91 across the four assessments, respectively.

### 2.4 Statistical analysis

Before data analysis, this study checked whether the data conforms to a normal distribution through skewness and kurtosis tests. The skewness range is -0. 81 to 2.20, with a kurtosis range of -1.11 to 4.81, indicating that the data exhibits approximately normal distribution characteristics (skewness<3, kurtosis<10; 47). In this study, a correlation analysis and repeated-measures ANOVA were performed for each variable using SPSS 26.0. Subsequently, a Gaussian graphical model (GGM) was constructed using the ‘qgraph’ package in R to estimate the PIU network structure. This study investigated the relationships between PIU symptoms and how these relationships change with age. Furthermore, comparisons of the PIU symptom networks across the four assessments and between genders were performed. The network analysis methods strictly followed the standard guidelines outlined by Epskamp et al. (48).

#### 2.4.1 Handling missing data

The study data were obtained from a three-year longitudinal tracking study conducted over four assessments. Owing to reasons such as student miss filling and other reasons, the data is incomplete, with a missing data rate of 0.92%, they can be considered negligible (49). Subsequently, the missing data were addressed using the ‘mice’ function in R for multiple imputation (50, 51). Multiple imputation is a method that estimates missing values by creating multiple complete datasets in which each dataset fills in the missing values with several possible replacement values.

#### 2.4.2 Confirmatory factor analysis

In this study, through the Mplus software, the Maximum Likelihood Robust estimation was used. Confirmatory factor analysis (CFA) was conducted in MLR to verify the validity of the factor structure. The goodness of fit of the model was evaluated by comparing indicators such as the Fitting index (CFI), the Tarker-Lewis index (TLI), the approximate root mean square error (RMSEA), and the standardized residual root mean square (SRMR). The fitting criterion was CFI>0.90. TLI>0.90, SRMR<0.06, RMSEA<0.08 (52).

#### 2.4.3 Network estimation

The partial correlation networks of PIU symptoms at four different time points were estimated and visualised using the qgraph package in R. Because PIU is a continuous variable, we used a GGM based on partial correlation analysis (21). GGM is a suitable method for non-binary data and assumes that all nodes are distributed normally. Network models were regularised using the extended Bayesian information criterion (53) with the least absolute shrinkage and selection operator (54) to shrink weak connections to zero, reduce spurious correlations, and enhance network interpretability (55). In plotting the grid plots using the qgraph package, the averageLayout function was used to ensure a consistent layout across the symptom networks at the four time points, facilitating visual comparisons. In the grid plots, the thickness of the edges indicates the strength of the correlations between two nodes: thicker blue edges represent positive correlations, and thicker red edges represent negative correlations.

#### 2.4.4 Centrality estimation

Central symptoms within a network are evaluated using centrality metrics, which typically include strength, closeness centrality, betweenness centrality and Expected Influence(EI) (56). Strength refers to the sum of the absolute values of the strength of a node’s connections with other nodes in the network. Closeness centrality is the reciprocal of the sum of the shortest path lengths between all nodes in the network. Betweenness centrality represents the frequency with which a node lies on the shortest paths between any two other nodes and is also considered a bridge connecting other symptoms. EI sums the raw weights of edges, taking the sign of the edge into account (57). Previous research has suggested that closeness and betweenness centrality can be unstable (58). In this study, most of the edge weights are positive, and the strength values are nearly equal to the Expected Influence (EI) values (See **Tables 1**–**4**). Therefore, this study primarily used strength as a centrality measure for the analysis (59, 60).

**Table 1:** GGM network weight matrix for different dimensions of the Problematic Internet Use Scale at T1 among junior high school students.

Items	Q1	Q2	Q3	Q4	Q5	Q6	Q7	Q8	Q9	Q10	Q11
Q1											
Q2	0.15										
Q3	0.00	0.14									
Q4	0.17	0.11	0.08								
Q5	0.00	0.17	0.00	0.20							
Q6	0.00	0.02	0.00	0.19	0.09						
Q7	0.00	0.00	0.00	0.09	0.10	0.22					
Q8	0.05	0.24	0.02	0.15	0.08	0.09	0.17				
Q9	0.00	0.00	0.16	0.03	0.19	0.11	0.08	0.08			
Q10	0.10	0.10	0.07	0.05	0.10	0.02	0.08	0.03	0.10		
Q11	0.08	0.03	0.06	0.00	0.00	0.02	0.16	0.05	0.15	0.17	

Q1: time passes quickly on the Internet; Q2: hard to control; Q3: decline in academia; Q4: being comfortable; Q5: feel bad mood; Q6:conceal the truth; Q7:fewer leisure activities; Q8: being conflicted; Q9: feeling depressed; Q10: feeling of missing; Q10: say/do what is not done in reality. The same below.

**Table 2:** GGM network weight matrix at T2.

Items	Q1	Q2	Q3	Q4	Q5	Q6	Q7	Q8	Q9	Q10	Q11
Q1											
Q2	0.15										
Q3	0.07	0.31									
Q4	0.04	0.03	0.14								
Q5	0.08	0.03	0.01	0.38							
Q6	0.00	0.00	0.00	0.12	0.09						
Q7	0.00	0.06	0.00	0.16	0.09	0.15					
Q8	0.10	0.25	0.00	0.15	0.10	0.15	0.07				
Q9	0.00	0.06	0.00	0.00	0.02	0.19	0.26	0.09			
Q10	0.02	0.00	0.00	0.20	0.10	0.00	0.10	0.00	0.20		
Q11	0.00	0.00	0.00	0.00	0.08	0.20	0.00	0.05	0.00	0.20	

**Table 3:** GGM network weight matrix at T3.

Items	Q1	Q2	Q3	Q4	Q5	Q6	Q7	Q8	Q9	Q10	Q11
Q1											
Q2	0.11										
Q3	0.09	0.21									
Q4	0.08	0.25	0.03								
Q5	0.05	0.00	0.02	0.28							
Q6	0.00	0.00	0.00	0.18	0.00						
Q7	-0.33	0.00	0.10	0.04	0.07	0.07					
Q8	0.02	0.24	0.13	0.01	0.10	0.23	0.16				
Q9	0.00	0.00	0.12	0.00	0.27	0.09	0.19	0.170.04			
Q10	0.10	0.00	0.00	0.15	0.13	0.00	0.04.064	0.04	0.17		
Q11	0.00	0.00	0.00	0.08	0.07	0.07	0.06	0.17	0.00	0.26	

**Table 4:** GGM network weight matrix at T4.

Items	Q1	Q2	Q3	Q4	Q5	Q6	Q7	Q8	Q9	Q10	Q11
Q1											
Q2	0.12										
Q3	0.14	0.24									
Q4	0.03	0.17	0.12								
Q5	0.08	0.08	0.06	0.34							
Q6	0.00	0.04	0.13	0.03	0.03						
Q7	0.00	0.00	0.00	0.00	0.00	0.18					
Q8	0.00	0.19	0.00	0.05	0.07	0.23	0.30				
Q9	0.00	0.00	0.08	0.11	0.10	0.02	0.23	0.26			
Q10	0.19	0.04	0.00	0.14	0.09	0.00	0.00	0.02	0.22		
Q11	0.00	0.00	0.00	0.00	0.14	0.18	0.07	0.02	0.02	0.2	

#### 2.4.5 Network accuracy and stability

Network accuracy and stability were assessed using the bootnet package (48). First, nonparametric bootstrap difference testing was conducted to examine node strength and edge weight differences. Second, the accuracy of the edge weights was estimated by calculating the 95% confidence intervals of the bootstrap edge weights. Smaller confidence intervals indicate a lower sampling variability of the edge weights, indicating a more accurate network estimation. Finally, the stability of the centrality estimates was evaluated using the correlation stability coefficient (CS-coefficient) through subset bootstrap procedures. A CS coefficient greater than 0.25 indicates acceptable stability, while a coefficient greater than 0.5 indicates good stability (48).

#### 2.4.6 Network comparison

The Network Comparison Test (61) is a method used to examine differences in various network characteristics. We implemented this approach using the NetworkComparisonTest package in R (62). The differences between the two networks were assessed based on network structure invariance, global strength invariance, and edge strength invariance. The NetworkComparisonTest package was utilised in this study to examine differences in PIU symptom networks across four time points and between genders. This involved conducting network comparisons to evaluate network differences.

## 3 Results

### 3.1 Demographic variable statistical results

This study surveyed a total of 302 junior high school students, with 136 males (45%) and 166 females (55%). The majority of students were not only children (73.8%) and came from rural or township areas (74.2%). Regarding parental occupation, the highest proportion of fathers and mothers were engaged in manual labor, construction, technical work, self-employment, and related fields, accounting for 54.3% and 38.4%, respectively. In terms of parental education, the highest proportion of both fathers and mothers had completed junior high school, accounting for 48.7% and 51%, respectively. Concerning family economic status, the largest proportion of students had a family annual income ranging from 50,000 to 100,000 RMB, representing 22.8%.

### 3.2 Confirmatory factor analysis results

This study used the Confirmatory factor analysis method to test the structural validity of the problematic Internet usage scale in four stages. The model data fit well. Specifically, in stage T1, CMIN/DF=1.588, CFI=0.963, TLI=0.954, RMSEA=0.044. SRMR=0.043. In the T2 stage, CMIN/DF=2.628, CFI=0.913, TLI=0.891, RMSEA=0.056, SRMR=0.073. In the T3 stage, CMIN/DF=2.417, CFI=0.928, TLI=0.909, RMSEA=0.047, SRMR=0.068. At the T4 stage, CMIN/DF=3.605, CFI=0.892, TLI=0.865, RMSEA=0.057, SRMR=0.057. The criteria were basically met (52), and the results indicated that the scale had good structural validity in all four stages.

### 3.3 Descriptive statistics, correlation analysis, and analysis of variance

As **Table 5** shows, the correlations among the four time points were statistically significant, with a significant positive correlation between T1 and T2 PIU scores (r=0.45, *P <*0.01), significant positive correlation between T2 and T3 (r=0.44, *P <*0.01), and significant positive correlation between T3 and T4 (r=0.51, P <0.01). A repeated-measures ANOVA was performed with PIU scores as the dependent variable and measurement time as the independent variable. The homogeneity of variance was not satisfied; therefore, the Huynh-Feldt correction was applied. After correction, F_(2.68, 806.94)_=39.88, *P*<0.01, considering significant differences in PIU scores across the four time points, and after pairwise comparison, statistical differences were observed at all four time points. Specifically, T1 < T2 < T3 < T4, meaning that the mean value of PIU increased annually from T1 to T4, these findings indicate that adolescents are more likely to develop an over-reliance on the Internet as they age and thus show more severe PIU symptoms.

**Table 5:** Descriptive statistics and correlative analysis results.

Variables	M	SD	T1	T2	T3	T4
T1	2.51	1.12	1			
T2	2.67	1.12	0.45**	1		
T3	2.97	1.16	0.25**	0.44**	1	
T4	3.28	1.24	0.16**	0.27**	0.51**	1

**p < 0.01. M, Mean; SD, Standard deviation.

### 3.4 Network estimation

**Figure 1:**
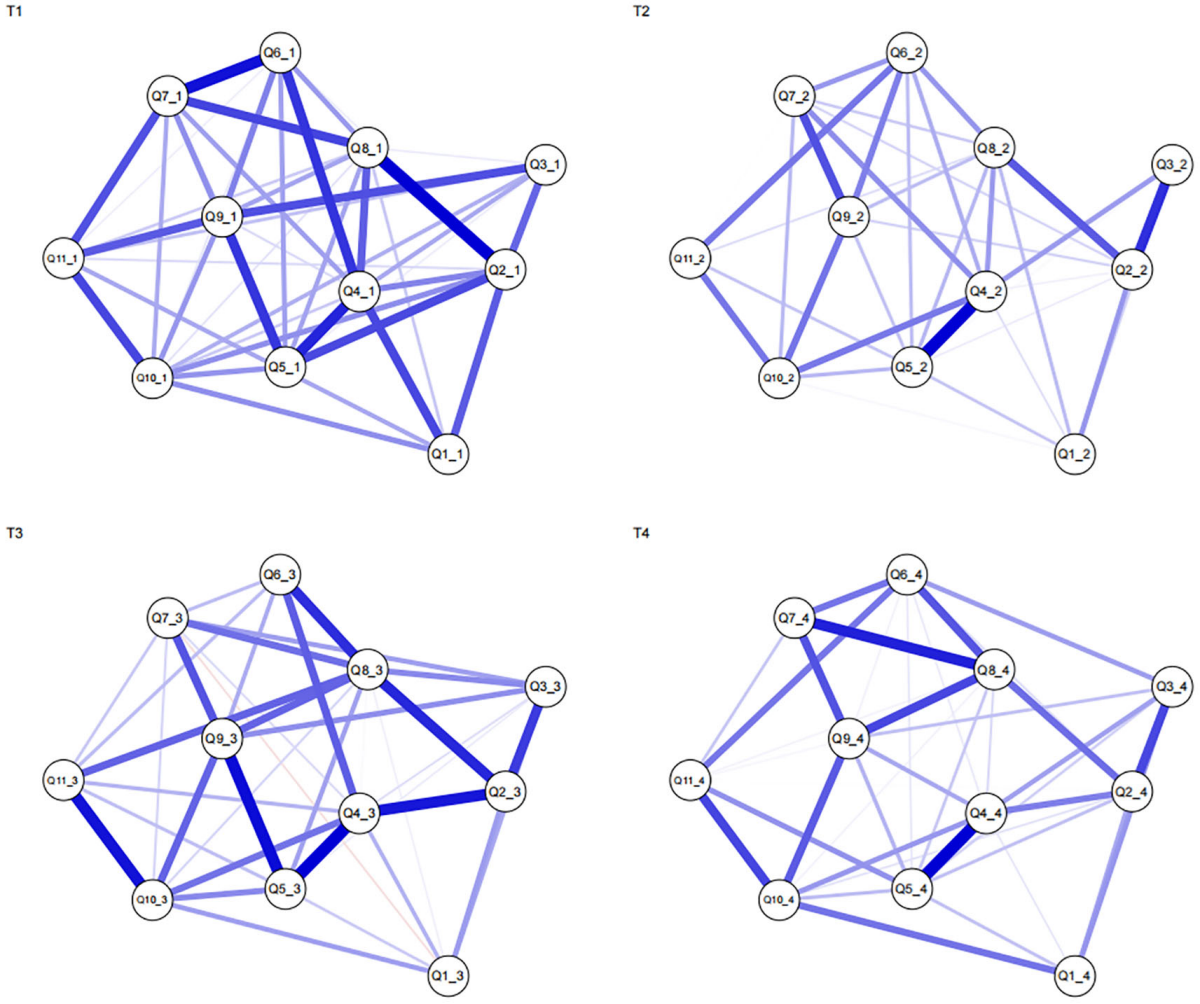
GGM Networks of PIU in Junior High School Students across Four Phases. In this figure, owing to the lengthy names of the 11 items in the Problematic Internet Use Scale, for ease of presentation, this paper uses the format Q1–Q11 to denote them, with the items from the first assessment labelled as Q1_1 and so on. Blue indicates positive correlations, whereas red indicates negative correlations. Q1: time passes quickly on the Internet; Q2: hard to control; Q3: decline in academia; Q4: being comfortable; Q5: feel bad mood; Q6:conceal the truth; Q7:fewer leisure activities; Q8: being conflicted; Q9: feeling depressed; Q10: feeling of missing; 10: say/do what is not done in reality. The same below.

The PIU symptom networks at the four time points are depicted in the **Figure 1**. Each network model comprised 55 edges (11 × (11-1)/2). Respectively, the proportions of non-zero correlations in the networks were 45/55, 37/55, 39/55, and 39/55, with average network densities of 0.82, 0.67, 0.71, and 0.71, respectively. At T1, the strongest correlation (0.24) was observed between nodes 2 (‘hard to control’) and 8 (‘being conflicted’). From T2 to T4, the highest correlations were found between nodes 4 (‘being comfortable’) and 5 (‘feel bad mood’), with coefficients of 0.38, 0.28, and 0.34 respectively. All edge weights across the four time points are listed in **Tables 1**–**4**.

### 3.5 Centrality estimation

**Figure 2:**
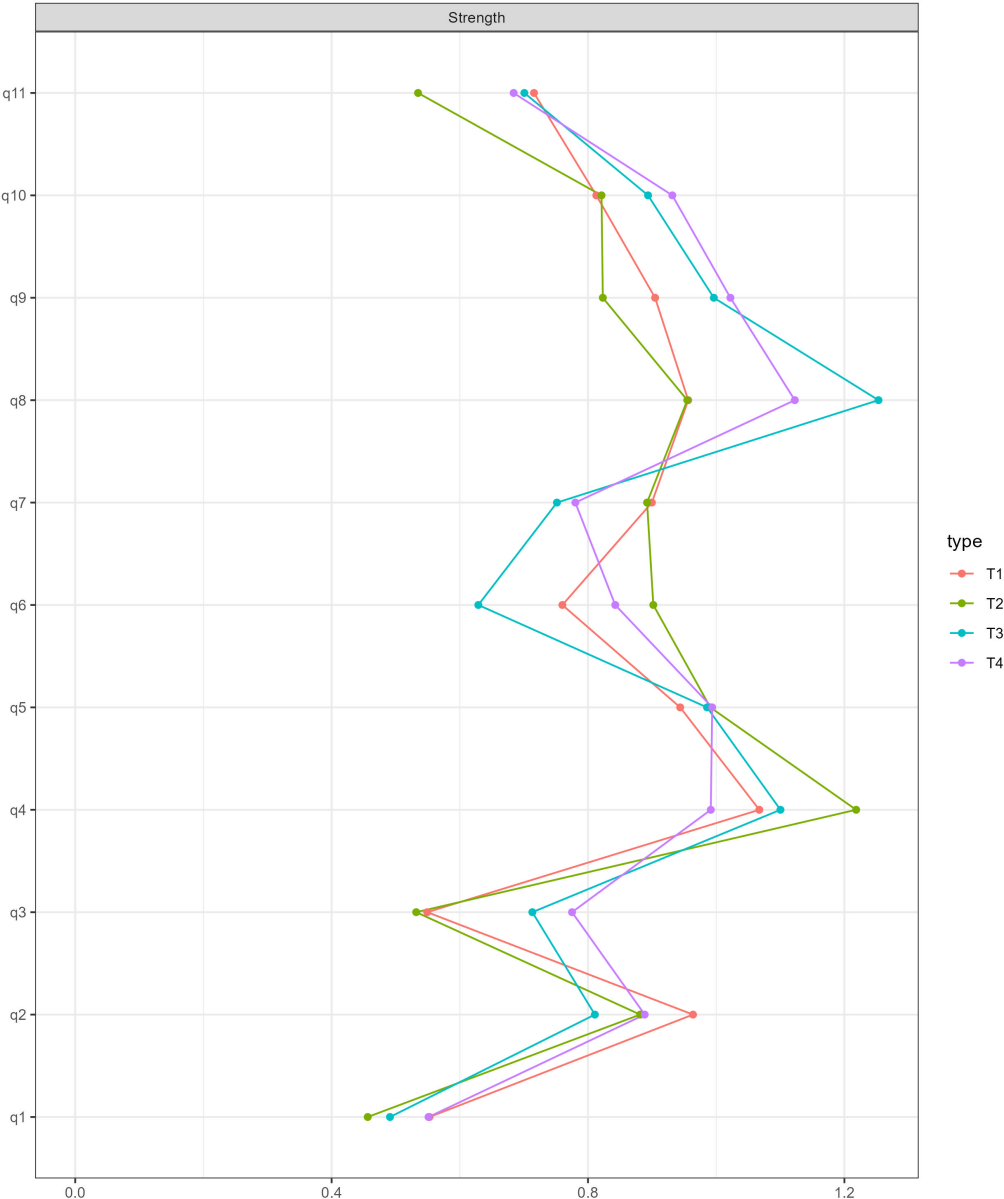
Centrality strength of networks at four time points.

The centrality measures at the four time points are presented in the **Figure 2**. At T1 and T2, node 4 (‘being comfortable’) exhibited the most prominent strength centrality. However, at T3 and T4, node 8 (‘being conflicted’) became the most central symptom, reflecting the deepening of PIU behaviours and highlighting the crucial roles of cognitive conflict and self-control failure in PIU development. Understanding these relationships is pivotal for understanding the dynamics of PIU. Overall, nodes 4, 5, and 8 consistently maintained high strength centrality from T1 to T4, collectively forming key nodes in the PIU relationship network.

### 3.6 Network accuracy and stability

**Figure 3:**
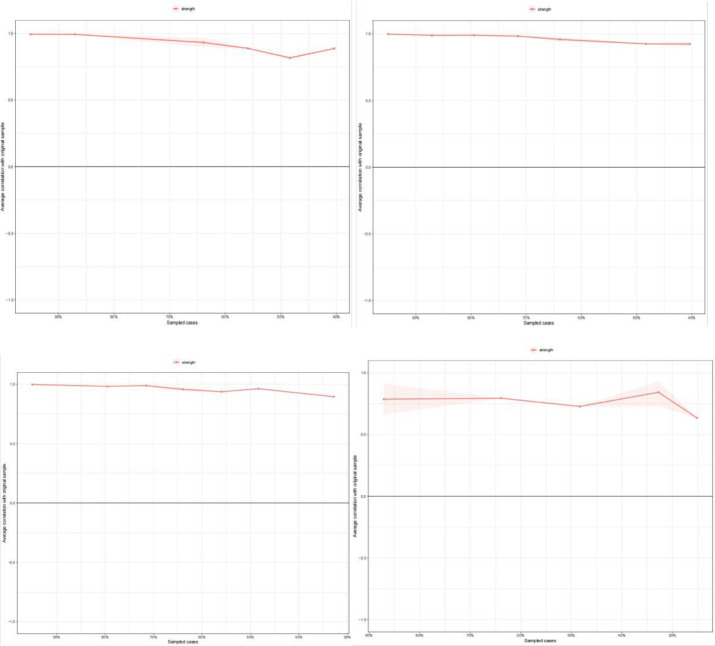
Centrality stability of the PIU symptom network.

Bootstrap difference testing was used to estimate the node strength and edge weight differences, as shown in **Supplementary Figure 1** and **Supplementary Figure 2**. According to the bootstrap edge weight structure (**Supplementary Figure 3**), the network estimates across the four phases for junior high school students were moderately accurate As the analysis results in **Figure 3** show, the CS-coefficients for strength centrality across the four phases were 0.596, 0.596, 0.672, and 0.672, indicating high stability.

### 3.7 Network comparison

As shown in **Table 6**, no significant differences were observed in the overall network structure and global strength among the networks across the four time points. Thus, the network structures of the PIU symptoms remained similar over time.The specific values of the global strength of the four-stage network are:T1=0.456, T2=0.450, T3=0.466, T4=0.479.

**Table 6:** Edge weights and overall strength of the networks at four time points.

Network comparison results	Network Structure		Network Global Strength	
	M	p-value	GS	p-value
T1vsT2	0.18	0.77	0.06	0.80
T1vsT3	0.17	0.92	0.10	0.58
T1vsT4	0.16	0.91	0.23	0.50
T2vsT3	0.25	0.18	0.16	0.26
T2vsT4	0.22	0.29	0.29	0.24
T3vsT4	0.17	0.74	0.13	0.58

We further explored the gender differences in the internal structure of PIU symptom networks among junior high school students. The results showed no significant differences in network structure or global strength based on gender (M=0.29, P=0.85; GS=3.8, P=0.83).

## 4 Discussion

This study is the first to use a network analysis approach to investigate the relationships among PIU symptoms in junior high school students over three years. This study also identified core symptoms at each stage and explored age-related differences. First, we delineated the connections between PIU symptoms across different stages of middle school. Second, we examined variations in PIU symptom networks across these four stages and between genders. These findings provide suggestions for targeted intervention strategies for preventing and addressing PIU among junior high school students.

### 4.1 Interconnections among PIU symptoms in junior high school students

At T1, nodes 2 (‘hard to control’) and 8 (‘being conflicted’) exhibited the highest edge weights in the network. The strong correlation between these two symptoms may affect the overall manifestation of PIU. This finding suggests that despite recognising the harm of prolonged Internet use, junior high school students struggle to control or reduce their online time (63). However, at T2–T4, the relationship between nodes 4 (‘being comfortable’) and 5 (‘feel bad mood’) emerged as the strongest in the network. This indicates that as junior high school students age, they become increasingly comfortable spending more time online, which is closely intertwined with their frustration when unable to access the Internet (64). Together, these factors were found to significantly influence PIU among junior high school students.

### 4.2 Core symptoms and evolution of PIU in junior high school students

At T1 and T2, node 4 (‘being comfortable’) exhibited the greatest strength. This symptom aligns with the diagnostic criteria for PIU (65), in which individuals experience withdrawal symptoms such as discomfort when reducing or stopping Internet use and an increasing need to spend more time and effort online to achieve satisfaction. This finding is consistent with those of Li and Chung (66) and Chen et al. (44), indicating that individuals require more Internet use to feel happy or satisfied and may experience distress when their Internet use is reduced or stopped. This core symptom reveals junior high school students’ over-dependence on and addiction to Internet use; therefore, we summarise node 4 as ‘being comfortable’. Adolescents entering middle school are in a critical physical and psychological development stage. They may feel unfamiliar and uncomfortable with new environments and relationships, leading them to invest more time and energy online to fulfil their various emotional needs (67, 68). The Internet can become a crucial avenue for seeking identity and belonging during this period. Restrictions or interruptions in Internet use can evoke strong discomfort, potentially having a negative effect on daily life and learning (8, 31).

As junior high school students aged, at T3 and T4, node 8 (‘being conflicted’) not only rapidly activates other PIU but symptoms also acts as a core symptom within the network. This node is highly consistent with the core behavioural addiction symptom of loss of control (69), which we summarise as ‘being conflicted’. This result is consistent with the behavioural addiction model proposed by Brand et al. (70), which emphasises the crucial role of prefrontal control processes in the development of Internet addiction and other behavioural disorders. Thus, loss of control occupies a central position within the network and is closely linked with other symptoms. With increasing age, junior high school students gradually recognise the adverse effects of prolonged Internet use on their academic performance, health, and social interactions. However, owing to the formation of PIU, their behaviour does not align with their cognition, indicating weakened control over Internet use (71). Particularly in the eighth and ninth grades, students face various pressures, such as entrance exams, making the Internet an escape from reality (72). This propensity increases their susceptibility to becoming excessively immersed in the Internet and finding it difficult to disengage.

The evolution of core symptoms from T1 to T4 indicates that in the initial stages of PIU, adolescents’ primary motivation for Internet use is seeking comfort and satisfaction. At this stage, Internet use serves as a temporary escape from the pressures, troubles, and boredom of adolescent life (73, 74). However, over time, junior high school students require increasing amounts of time to achieve satisfaction and comfort from Internet use, leading to a gradual increase in the frequency and duration of use, eventually becoming habitual. As dependence on the Internet deepens, adolescents begin to recognise the potential negative consequences, such as sleep deprivation, vision problems, and academic neglect (75). Despite this awareness, they struggle to control their Internet use behaviour, exacerbating the symptoms of PIU. In summary, the change in the core symptoms of PIU is an evolution from ‘being comfortable’ to ‘being conflicted’. Considering the challenging trajectory outlined above, early prevention and intervention for PIU among junior high school students may be crucial for promoting healthy adolescent development.

Based on the network analysis across four stages, the study found that three symptoms consistently exhibited high intensity: ‘being comfortable’, ‘feel bad mood’, and ‘being conflicted’. These symptoms are crucial diagnostic criteria for PIU (65, 76) and reflect addictive features. Adolescents may gradually develop a strong dependence on the Internet, using it to escape reality, relieve stress, or seek satisfaction. Consequently, they require increasing amounts of time online to maintain this satisfaction and experience discomfort or anger when unable to access the Internet. Despite having the correct cognitive awareness, adolescents often struggle with self-control (77) and potentially lack effective coping strategies for handling real-life issues. This difficulty may perpetuate a vicious cycle of excessive Internet use.

### 4.3 Differences in PIU networks by age and gender among junior high school students

In the network comparison test, no significant differences were observed in the overall network structure and global strength across the four time points, which is consistent with the findings of Liu et al. (37). This suggests that the network structure remained stable across these time points, with visual differences in network models, possibly due to variations in sample size or sampling effects (61). In previous studies (78), if network structures did not show significant differences in the two-group comparison tests, further detailed examinations of individual edge weights and node centrality were not conducted. Therefore, we did not delve deeper into these analyses, as the similarity in network structures across the four stages indicated that the significance and overall interactions of PIU symptom characteristics among junior high school students were stable and unaffected by age. That is to say, during the junior high school stage, the network structure of PIU symptoms has a certain degree of invariance and stability, which is consistent with the research results of Hirota et al. (35) and Liu et al. (37). Additionally, the non-significant increase in overall network strength across the four stages suggests that symptom connectivity remained unchanged (37). Meanwhile, from the first time point of measurement to the fourth time point, the global strength of the network did not reach a significant level, but the value increased. This suggests that the global strength of PIU in junior high school students may have a gradually increasing trend, that is, the symptom network may be denser or have stronger connections. In the future, further verification through longitudinal data is needed.

Research shows that there were no significant differences in network structure or edge weights between genders, and the interactions among PIU symptoms remained stable across genders. This is inconsistent with our hypothesis. Considering that previous studies have mostly focused on single symptoms of PIU (39), the network analysis indicators (network structure and global strength) used in this study, which pay more attention to overall connectivity, may mask gender differences in specific nodes (such as social interaction and gaming). That is, although there are differences between males and females in certain specific behaviors (e.g., duration of online socializing), the connection patterns between their symptoms may tend to be consistent. In the future, more detailed comparisons of local networks can be conducted through edge weight invariance tests (37, 79). In addition, the subjects of this study are junior high school students in early adolescence with a low level of PIU, and their Internet usage patterns may not have shown gender differentiation yet. It should be emphasized that the network invariance test used in this study is a data-driven approach that covers all nodes and edges of the network. Therefore, the findings of this study do not deny the existence of gender differences, but indicate that such gender differences may be less important when examining PIU (80).Therefore, interventions for PIU should not overly emphasise gender differences but rather focus on issues common to all junior high school students.

### 4.4 Implications and innovation

The results provide a scientific basis for developing different intervention strategies for PIU among junior high school students at various stages. In the early stages of middle school, the focus should primarily be on the core symptom of ‘being comfortable’. Existing research has shown that cognitive-behavioural therapy and music therapy can effectively reduce PIU behaviours in adolescents, thereby alleviating the psychological discomfort associated with reduced Internet use (81). Moreover, parents should set reasonable limits on Internet usage time and actively encourage adolescents to cultivate diverse interests and hobbies. Additionally, education and training programs on time-management skills may be effective measures (82). In the middle to late stages of middle school, addressing the issue of ‘being conflicted’ becomes particularly critical. Participation in sports has been proven to enhance self-control in adolescents (83). Therefore, personalised sports programs can not only improve adolescents’ self-control but also help reduce excessive reliance on the Internet (84). Furthermore, group psychological counselling has shown significant effects in alleviating academic pressure and PIU symptoms (85), suggesting it may serve as a viable intervention approach.

In general, this study has certain novelty. Previous studies have mostly focused on adolescents (mainly high school students) and college students. From the perspective of network analysis, this study further enriches the theoretical system in this field by tracking the network structure and core characteristics of junior high school students over four years. Secondly, the results of this study provide new approaches and insights for the intervention of problematic Internet use among junior high school students. The core symptoms of junior high school students over four years are different, and higher strength centrality has a greater impact on other symptoms in the network, which may provide evidence for formulating differentiated intervention measures based on the developmental stage of junior high school students. This also suggests that school administrators and clinical workers should include junior high school students’ problematic Internet use in students’ mental health files, regularly investigate students’ problematic Internet use, and establish a dynamic management mechanism.

### 4.5 Limitations and future studies

This study examined the evolution of symptoms of problematic Internet use in junior high school students over three years, providing important insights into the development of targeted interventions. Despite the novel and valuable nature of our study, the following limitations should be considered. First, this study relied on self-report questionnaires, which may be susceptible to social desirability bias. Future research could use multiple sources of information such as feedback from parents and teachers. Secondly, the participants in this study were only from one region in China, and most of them were non-only children, mainly from rural areas, with a single type of school involved, which limits the generalizability of our research results. Future research should expand the sampling scope by conducting surveys in a broader geographic area to enhance the representativeness of the sample and the generalizability of the findings. Future research should expand the sampling scope by conducting surveys in a broader geographic area to enhance the representativeness of the sample and the generalizability of the findings. Third, this study explored the symptom evolution of pathological Internet in junior high school students within three years, and the results can provide important practical significance for identifying and preventing problematic Internet use in junior high school students. Fourth, the current focus of this study is limited to single-variable analysis. But previous studies have shown that PIU is closely related to a variety of psychosocial factors. For example, emotions of adolescents, interpersonal relationships, personality traits, academic pressure, cognition, and family factors have all been confirmed to be closely related to the occurrence and maintenance of PIU (9, 86, 87), future studies could incorporate these psychological variables to explore symptom evolution and its causal relationship with PIU. Finally, Internet Gaming Disorder (IGD) has been classified as a mental illness in the DSM-5 and ICD-11. This study refers to this classification when describing the items of the PIU scale as “symptoms”. However, since PIU has not yet been included in the aforementioned authoritative diagnostic systems, caution should be exercised regarding whether the scale items can be strictly equated with the term “symptoms”. In addition, as there is no clear conclusion on the definition of PIU in the academic circle at present, the current symptom analysis of this study is based on the classification of measurement tool items. At the same time, considering the heterogeneity of PIU, that is, having different specific PIU behaviors (such as online game addiction, social network addiction, etc.). Whether the general symptom evolution pattern of PIU observed in this study can be generalized to other specific PIU behaviors remains debatable. Future research can conduct a more in-depth exploration of specific PIU behaviors to verify the conclusions of this study.

## 5 Conclusion

This study explored the network structure and symptom evolution in PIU among adolescents at the junior high school level, providing a new perspective for proposing more effective and comprehensive intervention measures. The study found that the core symptoms of PIU among junior high school students at T1 and T2 are ‘you need to spend more time online to feel comfortable’, while at T3 and T4, the core symptom shifts to ‘despite knowing that long-term Internet use is harmful, you cannot restrain yourself’. It reflects the evolution of PIU symptoms in junior high school students from ‘being comfortable’ to ‘being conflicted’. In addition, in the whole stage of junior high school, ‘the deepening of psychological dependence on the Internet’, ‘offline negative emotions’ and ‘cognitive and behavioural conflict’ have always maintained a high intensity centrality. Intervention strategies should focus on these aspects. Through education, cognitive behavioural therapy, and other means, adolescents can reduce their dependence on the Internet and adjust their negative emotions accordingly. In addition, we found that the PIU symptom network had a certain stability, and the interaction pattern between symptoms could remain unchanged for a long time, suggesting that we should maintain the continuity and stability of interventions to cope with the complexity and persistence of PIU in junior high school students. At the same time, the study found no significant gender differences in the PIU network, which also suggests that gender stereotypes should be avoided when conducting interventions and that more universally applicable intervention methods should be adopted.

## Data Availability

The raw data supporting the conclusions of this article will be made available by the authors, without undue reservation.
