# Supplementary material for: From comfortable to conflicted: a three-year longitudinal symptom evolution of problematic Internet use among junior high school students
Source: Front Psychiatry. 2025 Sep 5;16:1635911. doi: 10.3389/fpsyt.2025.1635911 (PMC12447571; doi:10.3389/fpsyt.2025.1635911)
Supplement: Supplementary file 1 [file Supplementaryfile1.docx]

**Supplementary information**


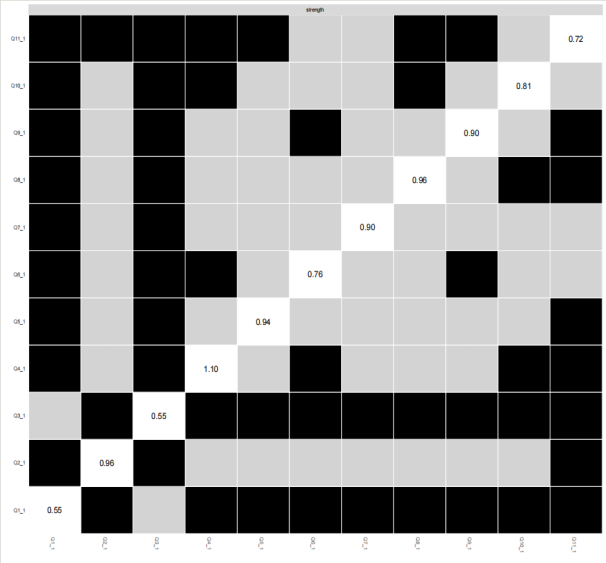

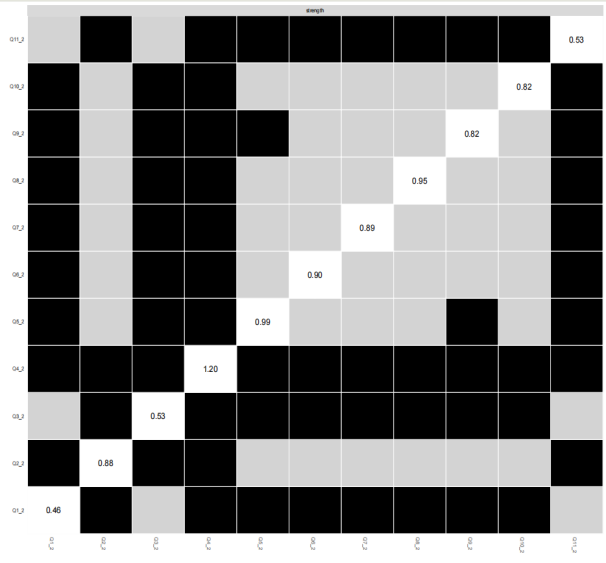


（T1） （T2）


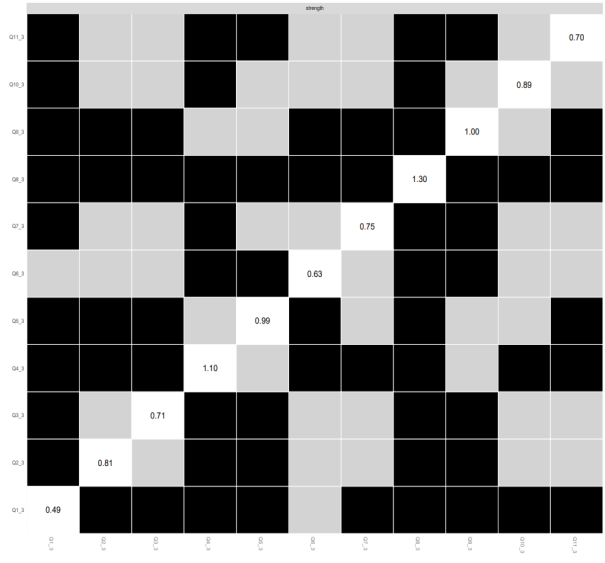

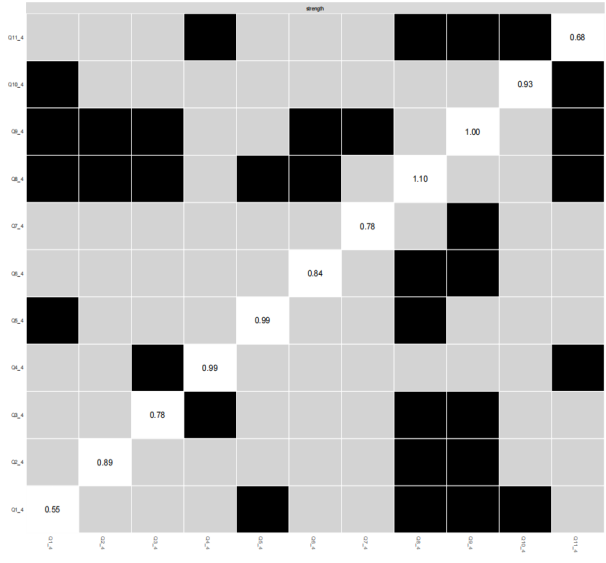


（T3） （T4）

**Supplementary Figure 1** Testing Differences in Centrality of Symptoms in PIU Network Nodes. Grey boxes indicate nodes or edges without significant differences, whereas black boxes indicate nodes or edges with significant differences. White boxes in the centrality plot represent node strength.


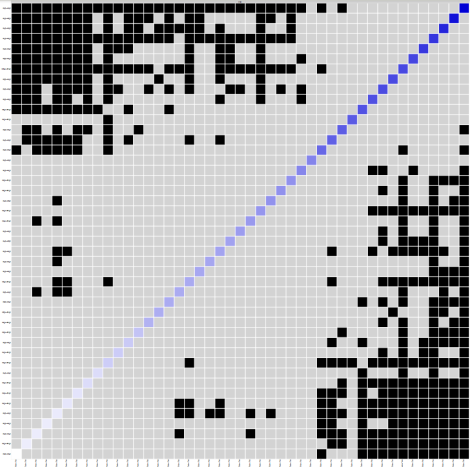

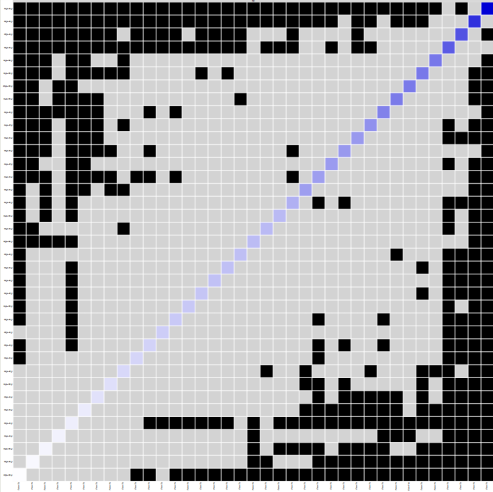


（T1） （T2）


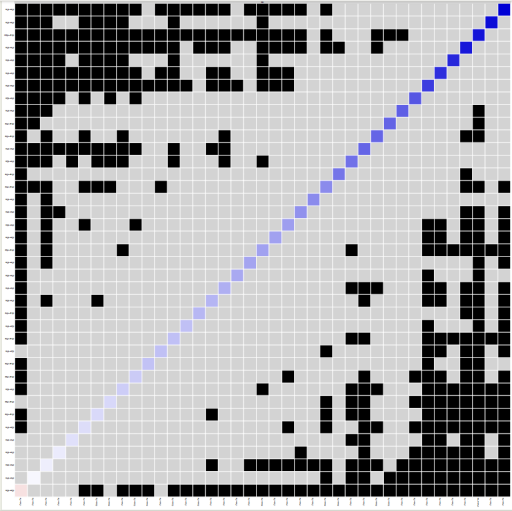

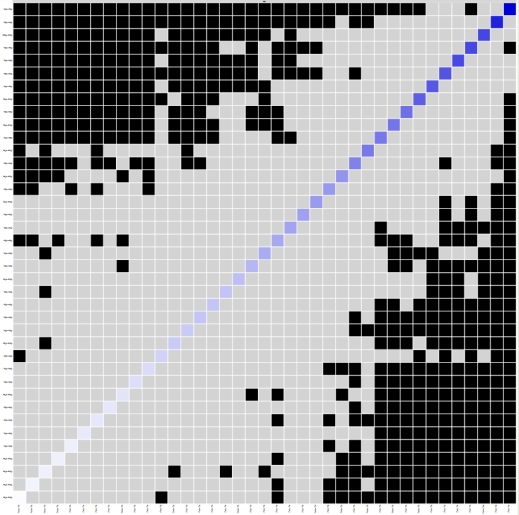


（T3） （T4）

**Supplementary Figure 2** Estimating Edge Weight Differences in PIU Symptom Self-help Variance Test.


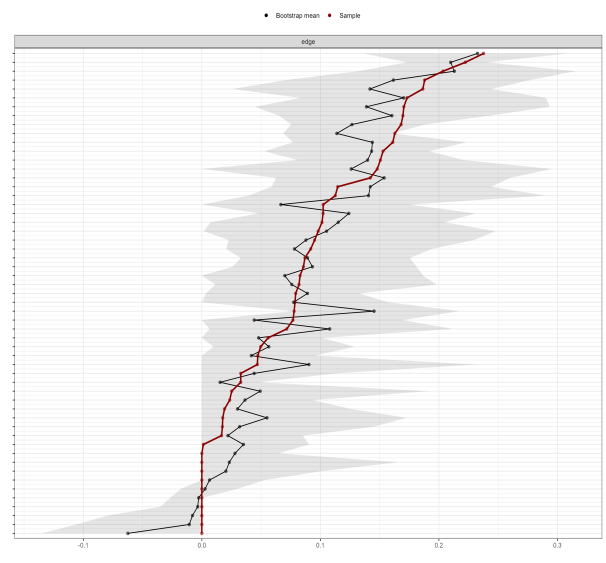

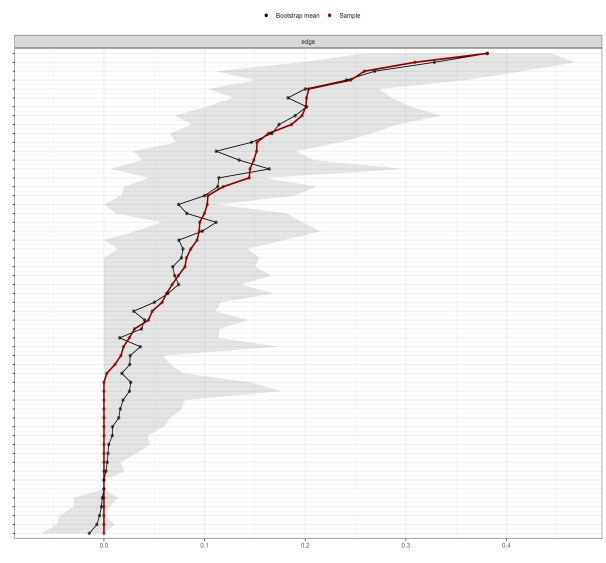


（T1） （T2）


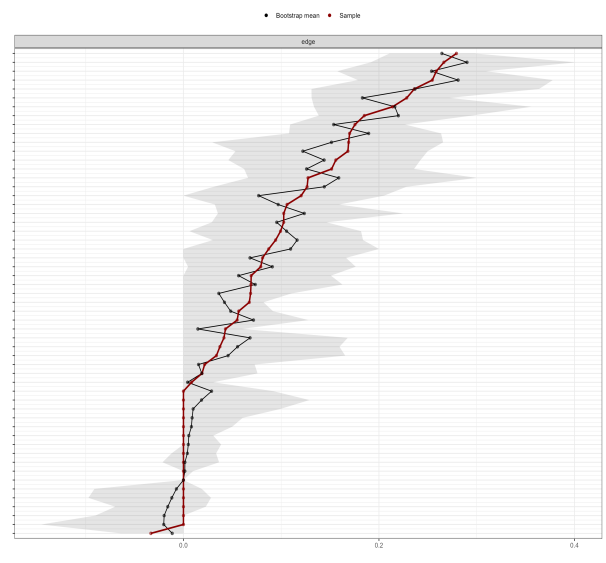

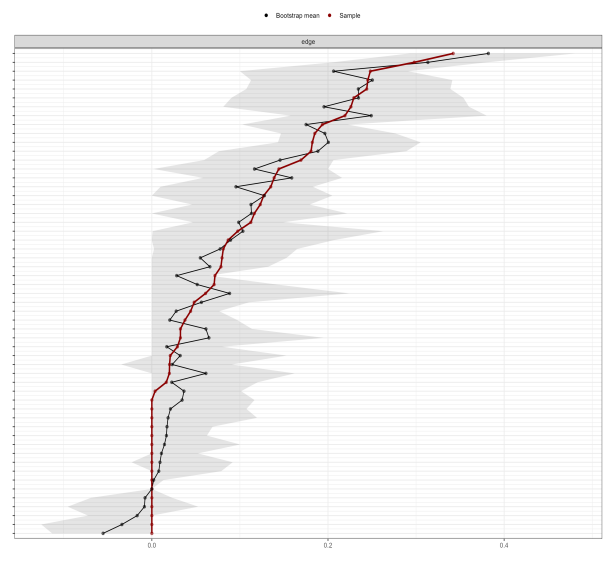


（T3） （T4）

**Supplementary Figure 3** Edge weight precision of the PIU symptom network (N = 302). Bootstrap confidence intervals are estimated for the edge weights of the network. The red line represents the sample value, and the grey areas indicate the bootstrap confidence intervals. Each horizontal line represents an edge in the network, sorted from the highest to the lowest weight.

**Supplementary Table 1** Descriptions of each PIU item.

| Label | Item | Abbreviation |
| --- | --- | --- |
| Node 1 (Q1) | When you are online, time seems to pass quickly | time passes quickly on the Internet |
| Node 2 (Q2) | You cannot control or reduce your internet usage time | hard to control |
| Node 3 (Q3) | Excessive internet use leads you to neglect assignments or achieve less than desired grades in exams | decline in academia |
| Node 4 (Q4) | You need to spend more time online to feel comfortable | being comfortable because of online |
| Node 5 (Q5) | You feel angry or in a bad mood when you cannot access the internet | feel bad mood |
| Node 6 (Q6) | You hide your online time from your parents or family members | conceal the truth |
| Node 7 (Q7) | You have given up activities you used to be interested in (such as hobbies, sports, etc.) in order to be online | fewer leisure  activities |
| Node 8 (Q8) | Despite knowing that long internet use is harmful to you, you cannot control yourself | being conflicted |
| Node 9 (Q9) | Not being online for several hours makes you feel upset | feeling depressed |
| Node 10 (Q10) | You feel like you might miss out on important things if you are not online | feeling of missing |
| Node 11 (Q11) | You say or do things online that you would not do in real life | say/do what is not done in reality |
